# Early Effects of Elexacaftor-Tezacaftor-Ivacaftor Therapy on Magnetic Resonance Imaging in Patients with Cystic Fibrosis and Advanced Lung Disease

**DOI:** 10.3390/jcm11154277

**Published:** 2022-07-22

**Authors:** Letizia Macconi, Valeria Galici, Marco Di Maurizio, Enrica Rossi, Giovanni Taccetti, Vito Terlizzi

**Affiliations:** 1 Department of Radiology, Meyer Children’s Hospital, 50139 Florence, Italy; letizia.macconi@meyer.it (L.M.); marco.dimaurizio@meyer.it (M.D.M.); enrica.rossi@meyer.it (E.R.); 2 Department of Paediatric Medicine, Cystic Fibrosis Regional Reference Center, Meyer Children’s Hospital, 50139 Florence, Italy; valeria.galici@meyer.it (V.G.); giovanni.taccetti@meyer.it (G.T.)

**Keywords:** diffusion-weighted magnetic resonance imaging, *CFTR* modulators, score

## Abstract

This is a prospective, observational study involving three Cystic Fibrosis (CF) adult patients, evaluating the changes in chest magnetic resonance imaging (MRI) three months after the start of elexacaftor/tezacaftor and ivacaftor therapy. MRI showed a drastic reduction in mucus plugging and bronchial wall thickening, with an improvement in the diffusion-weighted MRI score. Similarly, a marked improvement in spirometric parameters, nutritional status, and sweat chloride was observed. Our preliminary data confirm that chest MRI could be a useful tool to assess disease progression in CF patients on modulatory drug therapy.

High-resolution computed tomography is currently the most sensitive tool to detect and monitor structural lung changes in Cystic Fibrosis (CF) [1]. Chest magnetic resonance imaging (MRI) is less sensitive to detecting structural changes and its image analysis systems are relatively poorly developed, however, it has better tissue differentiation allowing for the identification of mucus and study of functional aspects related to CF lung disease such as perfusion and ventilation. In addition, chest MRI can be used for further short-term follow-up study as its use is not restricted by ionizing radiation [2,3].

To date, few data are available evaluating the effect of *CFTR* modulators on MRI. In 2017, Altes et al. evaluated the effect of short- and long-term ivacaftor (IVA) treatment on hyperpolarized 3He-MRI in a cohort of CF patients aged ≥12 years with a G551D-*CFTR* mutation. The primary outcome was change from baseline in total ventilation defect; IVA treatment was effective in improving the local and overall lung ventilation [4].

Graeber et al. showed the beneficial effects of lumacaftor-IVA on lung ventilation, morphology, and perfusion in 30 Phe508del homozygous CF patients, 8–16 weeks after initiation of the therapy. Furthermore, MRI was more sensitive than forced expiratory volume in the 1st second (FEV_1_) in detecting a response to *CFTR* modulator therapy [5]. Recently, improvements in chest MRI after 4 [6] or 8–16 weeks [7] of Trikafta^®^ (a triple combination of two correctors, Elexacaftor (ELX) and Tezacaftor (TEZ)), together with the potentiator IVA, were reported in adults with CF, demonstrating the reversibility of structural lung and paranasal sinus abnormalities in patients with established disease [6,7].

Our paper is a prospective, observational study evaluating the early effects of ELX/TEZ/IVA on anatomical structure using MRI scores in three unselected adult CF patients (two females and one male; *CFTR* genotype: F508del in trans with N1303K, R553Q, and L1065P, respectively; age at the first chest MRI of 32, 43, and 20 years), followed at the CF center of Florence, Italy. The patients were enrolled in an ELX/TEZ/IVA managed access program, performed in Italy for CF patients aged over 12 years, heterozygous for the Phe508del variant and a minimal function variant, and with advanced lung disease {FEV_1_ < 40% in the preceding three months} [8]. Throughout the study, all patients continued their pre-study medications, including dornase alfa and respiratory physiotherapy.

The main outcome measure was to evaluate the effects of ELX/TEZ/IVA therapy on chest MRI scores {mBrody and diffusion-weighted magnetic resonance imaging (DWI)}, assessed in stable clinical conditions one month before and three months after the start of therapy. Scores are reported as means of the individual scores, obtained by two independent and blinded expert radiologists [3,9].

The secondary outcomes were the changes from baseline in sweat chloride concentrations (SCC), FEV_1_ and body mass index (BMI) after 3 months of therapy.

MRI were acquired in 20 min without the use of intravenous contrast or sedation and were based on T2-weighted sequences, PD-weighted sequences, and T2 STIR sequences (all sequences were MultiVane XD in order to minimize movements and cardiac artifacts), and diffusion weighted imaging with background suppression (DWIBS) with four b-values (0–200–400–800 s/mm^2^) acquired in free breathing using a clinical 1.5T MR scanner (1.5 Tesla Achieva D-Stream, Philips Medical Systems, the Netherlands).

The diffusion score was calculated by quantitative analysis of the signal intensity of the lesions. It was classified using a visual assessment of hypointensity, moderate intensity, or hyperintensity for each lobe, compared with the spinal cord, as in a previous study [3,9]. The global score resulted from the sum of the morphology and diffusion score.

The study was approved by the local ethics committee (Meyer Children’s Hospital, number 77/2019), and informed written consent was obtained from the involved adult patients.

The MRI exams performed after the therapy showed a drastic improvement of structural and moreover functional aspects of lung parenchyma (Figure 1). We emphasize the reduction in the DWI score for each lobe in the intensity and extension of the lesions (from 26 to 10, 30 to 13, and 48 to 13, respectively). Structural changes regarded all the evaluated parameters, and particularly the reduction in mucus plugging, with a cleansing of bronchiectasis, and reduction in bronchial wall thickening and the extent and the number of consolidation. Similarly, improvements were also found in the SCC (from 86 to 53 mEq/L, 112 to 35 mEq/L, and 123 to 20 mEq/L, respectively), FEV_1_ (from 31% to 62%, 36% to 45%, and 25% to 47%, respectively) value, and BMI (from 21.01 to 21.54, 24.78 to 25.19, and 17.47 to 18.73, respectively) (Table 1).

These cases confirm that MRI can detect abnormal lung structure and is sensitive to therapy response; furthermore, the use of DWI sequences allows for obtaining information about inflammation and structure without a contrast medium.

However, our study does have some limitations: as only three patients were included, no statistical analysis of the MRI score has been performed, limiting the scope of this preliminary study. For the same reason, the study could be subject to bias as the observers may have recognized the typical patterns belonging to a specific patient.

In conclusion, our data confirm the usefulness of MRI for assessing disease progression in CF patients on modulatory drug therapy. Given the increased survival of CF patients, increasing the use of MRI as a tool for the follow-up of CF patients is conceivable.

## Figures and Tables

**Figure 1:**
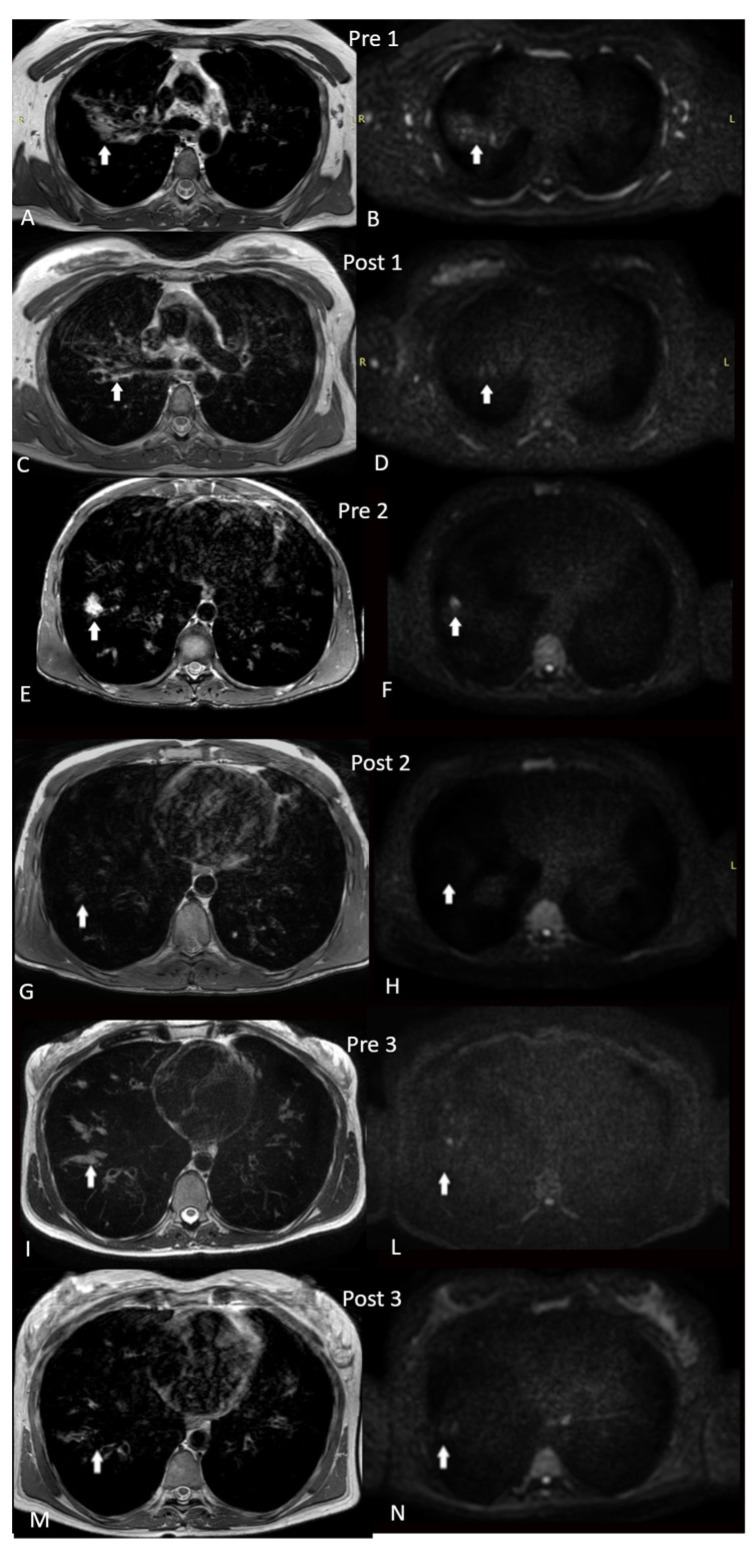
Patient 1, MRI images pre ELX/TEZ/IVA therapy: axial PD weighted image showing cilindric bronchiectasis with large amount of mucus plugging within and bronchial wall thickening (**A**). Atelectasis visible in the dorsal segment of the right upper lobe (**A**); DWIBS sequence at 800 b-value, with evidence of signal restriction in the mucus plugging (**B**). Patient 1, MRI images after 3 months of ELX/TEZ/IVA therapy: reduction in mucus plugging, and bronchial wall thickening (**C**); on DWIBS sequence at 800 b-value, no evidence of signal restriction (**D**). Patient 2, MRI images pre ELX/TEZ/IVA therapy: T2 axial image showing consolidation in the lateral segment of lower right lobe (**E**), with signal restriction in 800 b-value DWIBS (**F**). Patient 2, MRI images after 3 months of ELX/TEZ/IVA therapy: T2 axial image showing no consolidation (**G**) and no restriction in DWIBS sequence (**H**). Patient 3, MRI images pre ELX/TEZ/IVA therapy: PD weighted image, axial, showing evidence of mucus plugging on lateral segment of right lower lobe with bronchial wall thickening (**I**). Axial 800 b-value DWIBS sequence, showing signal restriction within the large mucus plugging (**L**). Patient 3, MRI images after 3 months of ELX/TEZ/IVA therapy: PD weighted axial, evidence of detersion of large airways mucus plugging of the bronchiectasis of the lateral segment of the lower right lobe, reduction in bronchial wall thickening (**M**). No evidence of signal restriction at 800 b-value DWIBS at the same level (**N**). Arrows were used to indicate the areas of interest.

**Table 1:** Demographic information and outcome measures at baseline and 3 months after Elexacaftor-Tezacaftor-Ivacaftor therapy.

Characteristics	Baseline	After 3 Months	Baseline	After 3 Months	Baseline	After 3 Months
	*Subject 1*		*Subject 2*		*Subject 3*	
*CFTR* genotype	F508del/N1303K		F508del/R553Q		F508del/L1065P	
Age (years)	32		43		20	
Sex	F		F		M	
Sweat chloride (mEq/L)	86	53	112	35	123	20
FEV_1_ (%)	31	62	36	45	25	47
BMI (kg/m^2^)	21.01	21.54	24.78	25.19	17.47	18.73

Abbreviations: *CFTR*: Cystic Fibrosis Transmembrane Regulator; FEV_1_: Forced Expiratory Volume in the 1st second; BMI: body mass index.

## Data Availability

Not applicable.

## Abbreviations

MRI	magnetic resonance imaging
ELX	elexacaftor
TEZ	tezacaftor
IVA	ivacaftor
PD	proton-density
DWIBS	diffusion weighted imaging with background suppression
